# *In Vitro* Evaluation of Antimicrobial Efficacy of Extracts Obtained from Raw and Fermented Wild Macrofungus, *Lenzites quercina*

**DOI:** 10.1155/2015/106308

**Published:** 2015-10-29

**Authors:** Olusola Clement Ogidi, Victor Olusegun Oyetayo, Bamidele Juliet Akinyele

**Affiliations:** Department of Microbiology, The Federal University of Technology, PMB 704, Akure, Ondo State, Nigeria

## Abstract

In recent time, there is a major concern about antibiotic resistance displayed by some pathogenic microorganisms and this had involved a continuous search for natural antimicrobial products. The phytochemistry as well as antimicrobial activity of extracts obtained from *Lenzites quercina* was investigated. The extracts and purified fractions were, respectively, tested against indicator organisms using agar well diffusion and disc diffusion methods. The quantity of phytochemicals found in the extracts of *L. quercina* ranged from 14.4 to 20.7 mg/g for alkaloids, 6.1 to 12.8 mg/g for steroids, 4.5 to 10.6 mg/g for saponins, 2.8 to 17.2 mg/g for terpenoids, and 0.41 to 17.1 mg/g for flavonoids. The gas chromatography mass spectrophotometry (GCMS) analysis of the extract reveals the presence of caprylic acid, stearic acid, tetradecanoic acid, methyl-11-octadecenoate, oleic acid, and 4-methyl-2-propyl-1-pentanol. Extracts of *L. quercina* and its purified fractions exhibited wider range of inhibition (4 mm to 26 mm) on *Staphylococcus aureus* (ATCC 29213), *Pseudomonas aeruginosa* (ATCC 27853), *Escherichia coli* (ATCC 35218), Methicillin Resistant *Staphylococcus aureus* (MRSA), *Salmonella typhi, Bacillus cereus, Enterococcus faecalis, Candida albicans*, and *Aspergillus niger*. The antimicrobial effects of *L. quercina* extracts indicate that this wild macrofungus contains significant amount of pharmacological agents, which could be extracted to curb the menace of antibiotic resistances by pathogenic organisms.

## 1. Introduction 

The current resistance of disease-causing organisms to common antibiotics is of serious concern and hence requires prompt attention. Species of* Pseudomonas*,* Escherichia coli*,* Enterococcus faecalis*,* Staphylococcus aureus*,* Shigella dysenteriae,* and* Salmonella typhi* had been termed as multidrug resistance organisms [1, 2]. The virulent potentials and drug-resistant patterns of these pathogenic organisms in community health settings are worrisome as most of these resistant bacteria are capable of horizontal gene transfer, decreased cell permeability against convectional antibiotics, and alteration of the ribosomal binding site [3]. Thus, the problem of antibiotic resistance is now a global challenge that is growing at an alarming pace but more rapidly in both underdeveloped and developing countries [4, 5].

Aside from the abuse of antibiotics, several circumstances such as production of antibiotics with lower active ingredient, self-medication, and sales of expired antibiotics contribute to the rapid spread of antibiotic resistance. In order to curb the problem of antibiotic resistances, the use of biologically active compounds from natural products needs to be explored. Medicinal mushrooms are richer source of natural secondary metabolites that are antimicrobial in nature [6]. The health-promoting benefits of secondary metabolites sourced from medicinal mushrooms had tremendously increased the chances of obtaining novel and safe antimicrobial compounds, which will combat and reduce the incidence of antibiotic resistance [7].

*Lenzites quercina* is basidiomycetes causing white rot in woods. This macrofungus is cosmopolitan in nature. However, there is dearth of information on the phytochemistry and antimicrobial properties of this macrofungus sourced in Akure, Ondo State, Nigeria. The present study is therefore meant to assess the phytochemical constituents and antimicrobial activity of raw and fermented* L. quercina* extracts.

## 2. Materials and Methods

### 2.1. Collection and Identification of Fruiting Body

Samples of* Lenzites quercina* were collected in November 2012 from farmland, in a nearby forest at The Federal University of Technology Akure, Nigeria (Latitude 07°14′N; Longitude 05°11′E). Sample of fruit body was morphologically identified and confirmed by molecular tools using the internal transcribed spacer (ITS) region of the rDNA.

### 2.2. Preparation and Percentage Yield of Extracts

*L. quercina* samples were divided into two portions. One portion was prepared as raw sample and the second portion was solidly fermented for four days at room temperature (27 ± 1°C). Both of the samples were dried and ground to powder using a mill machine (Retsch GmbH 5657 HAAN). The powders of the raw and fermented samples of* L. quercina* (100 g) each were sequentially extracted using 500 mL of 95% of petroleum ether, ethyl acetate, and ethanol, respectively. The filtrates obtained were dried using rotary evaporator (RE-52A, UNION Laboratories, England) and designated as RPP, raw* Lenzites quercina* extracted with pet-ether, REA, raw* Lenzites quercina* extracted with ethyl acetate, RET, raw* Lenzites quercina* extracted with ethanol, FPP, fermented* Lenzites quercina *extracted with pet-ether, FEA, fermented* Lenzites quercina *extracted with ethyl acetate, and FET, fermented* Lenzites quercina *with ethanol. All the reagents used during this study were of good analytical grade. The percentage yield of the extract was calculated as follows:

(1) Yield%=Weight of extract after dryingWeight of milled mushroom soaked×100.

### 2.3. Collection of Organisms

Microbial strains,* Staphylococcus aureus* (ATCC 29213),* Escherichia coli* (ATCC 35218),* Pseudomonas aeruginosa* (ATCC 27853),* Shigella flexneri* (ATCC12022),* Salmonella enterica* (ATCC 33458),* Bacillus subtilis* (ATCC 6633), and* Enterobacter aerogenes* (ATCC 13048),* Shigella flexneri*,* Salmonella typhi*,* Pseudomonas aeruginosa*,* Bacillus cereus*,* Enterococcus faecalis*,* Escherichia coli*,* Staphylococcus aureus*, Methicillin Resistant* Staphylococcus aureus* (MRSA),* Candida albicans*,* Aspergillus niger,* and* Aspergillus flavus,* were collected from Medical Microbiology Laboratory, University College Hospital (UCH), Ibadan, Oyo State, and Ondo State Specialist Hospital, Akure, Ondo State. The clinical isolates have prior history of resistance to some commercial antibiotics. The resistance pattern of MRSA used in the study was interpreted according to the criteria of [8]. The organisms were maintained on agar slant at 4°C and biochemically confirmed to ascertain their purity.

### 2.4. Assessment of Phytochemical Constituents in Extracts of* Lenzites quercina*

Qualitative and quantitative phytochemical analysis of* Lenzites quercina* extracts were carried out to assess the presence of flavonoid, tannins, saponins, alkaloids, phlobatannins, cardiac glycosides, steroids, and terpenoids using the methods of [9, 10]. The extracts were partially purified by using column and thin layer chromatography according to the methods of [11].

### 2.5. GC/MS Analysis of the* Lenzites quercina* Extracts

The bioactive compounds in the extract were identified with aid of gas chromatography mass spectrometry (QP2010 plus Shimadzu, Japan), which was equipped with a split injector and an ion-trap mass spectrometer detector together with a fused-silica capillary column having a thickness of 1.00 *μ*m, dimensions of 20 m × 0.22 mm, and temperature limits of 60°C to 325°C. The column temperature was programmed between 60°C and 250°C at a rate of 3.0 mL/min with pressure of 100.2 kPa. The temperature of the injector and detector was 250°C and 200°C, respectively. Helium gas was used as a carrier gas at flow rate of 46.3 cm/sec. The MS analysis was done based on comparative retention times, mass, and peaks of the chemical compounds using the computer-aided matching of unknown mass spectra of compounds with the known compounds stored in the software database library from the National Institute of Standards, Washington, USA, having more than 62,000 patterns as the reference database. This library enables the facilitation of comparison of generated spectra with the standards using Probability Based Matching algorithms. The GC-MS had been prefitted with a set of automated internal validity programmes for the analysis, including the adjustment of retention time function, scan measurement, and accurate compound identification from chromatogram, whose search is based on mass spectra similarity and other quality assurances or quality functions. The name, molecular weight, and structure of the components of the test materials were ascertained.

### 2.6. Antimicrobial Activity of Extracts and Fractions Obtained from* Lenzites quercina*

Agar well diffusion method of [12] was adopted for assessing the antimicrobial activity of the extracts. Briefly, the organisms were further cultivated on nutrient broth at 37°C for 24 hours and 28°C for 48 hours for bacteria and fungi, respectively. The inoculum size was adjusted to 0.5 McFarland turbidity standards. A sterile cotton swab was aseptically used to transfer organism on the dried surface of sterile Mueller Hinton Agar plate. Cork borer was used to make holes of 6 mm and extracts were aseptically applied. Commercial antibiotics such as ciprofloxacin, vancomycin, and fluconazole were used as positive control while sterile distilled water was used as negative control. Agar disc diffusion method of [13] was applied to assess the antimicrobial activity of the fractions obtained from the most effective extracts. The experiment was carried out in triplicate and inhibition zones were measured and recorded in millimeter.

### 2.7. Determination of Minimum Inhibitory Concentration (MIC) and MBC

The concentration of extracts was varied from 3.125 to 50 mg/mL and mixed with sterile nutrient broth with 0.1 mL of standardized inoculum (0.5 McFarland turbidity standards) in test tubes [13]. The tubes containing bacterial isolates were incubated aerobically at 37°C for 24 hours while the fungi isolates were incubated at 28°C for 48 hours. The test tubes containing the growth medium, sterile distilled water, and inoculum of each organism were maintained as control. The lowest concentration of the extract that produced no visible growth (no turbidity) when compared with the control tubes was regarded as MIC. The minimum bactericidal concentration (MBC) was determined by quantitatively subculturing 0.1 mL from test tube on Mueller Hinton Agar (MHA). These plates were incubated at 37°C. The MBC is defined as the lowest concentration of extracts that kill the bacteria tested at 99.9 to 100%.

### 2.8. Statistical Analysis

Data obtained were analyzed by one way analysis of variance and means were compared by Duncan multiple range test (SPSS 17.0 version). Differences were considered significant at *P* ≤ 0.05.

## 3. Results

**Figure 1:**
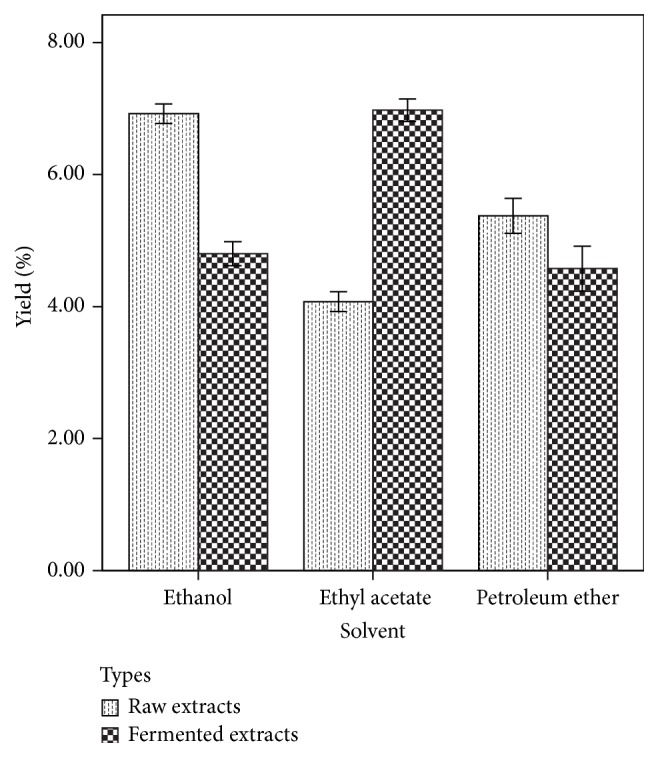
Percentage yield of extracts obtained from raw and fermented* Lenzites quercina *with different solvents.

**Figure 2:**
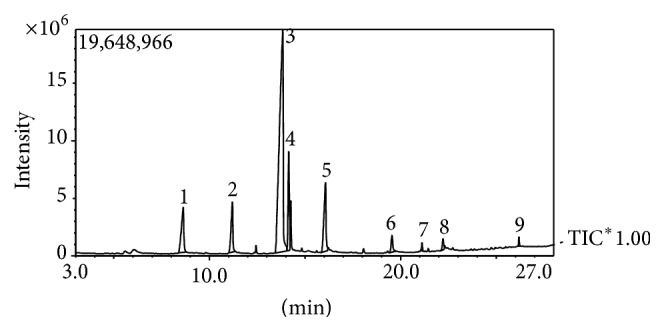
GC/MS analysis showing peaks of detected metabolites in ethanolic extract of* Lenzites quercina.*

Figure 1 shows the percentage yield of the extracts obtained from* Lenzites quercina.* Ethyl acetate and ethanol have the highest percentage yield of 7.8 and 7.7% for fermented and raw* Lenzites quercina, *respectively.  Table 1 shows the result of phytochemical properties of the extracts. The value obtained was 14.4 to 20.7 mg/g for alkaloids, 6.1 to 12.8 mg/g for steroids, 4.5 to 10.6 mg/g for saponins, 2.8 to 17.2 mg/g for terpenoids, and 0.41 to 17.1 mg/g for flavonoids.  Table 2 shows the bioactive compounds identified in the fractions of the extracts. The GCMS analysis of the extract revealed the presence of caprylic acid, stearic acid, tetradecanoic acid, methyl-11-octadecenoate, oleic acid, and 4-methyl-2-propyl-1-pentanol.  Figure 2 shows the peak of bioactive compounds present in* Lenzites quercina.* Tables 3 and 4 show the inhibited zones of extracts and fractions against tested isolates. The inhibition zones ranged from 4 mm to 26 mm. Tables 5 and 6 show the minimum inhibitory concentration and minimum bactericidal concentration. The MIC value ranged from 3.125 to 50 mg/mL and MBC ranged from 3.125 to 100 mg/mL.

## 4. Discussion

There are appreciable quantities of natural bioactive compounds in medicinal mushrooms. These compounds can be isolated and synthesized into new drugs to complement commonly used antibiotics. Therefore, there is need to explore the medicinal potentials of many yet untapped wild macrofungi that may be able to inhibit the resistance posed to antibiotics by pathogenic microorganisms. This study provides information on the phytochemical constituents, bioactive compounds, and antimicrobial efficacy of extracts obtained from a wild macrofungus,* Lenzites quercina.*

Qualitative and quantitative screening of the extracts of* Lenzites quercina* revealed the presence of flavonoid, terpenoids, saponins, alkaloids, and tannins. Maafi et al. [14] had earlier reported these phytochemicals in some widely available and commonly utilized macrofungi such as* Pleurotus *sp.,* Ganoderma lucidum*, and* Lentinus edodes*. The quantity of alkaloids (6.1 to 12.8 mg/g) in* Lenzites quercina* extract is in line with Ribeiro et al. [15] who had reported such range for some wild edible mushrooms.

The presence of stearic and oleic acid (Table 2) in the extracts of* Lenzites quercina* conformed to findings of Bisen et al. [16] who had revealed some fatty acids in* Lentinus edodes* as a component that is responsible for its pharmacological actions. Extracts of* Lenzites quercina* contain caprylic acid, which has been reported as natural fatty acid with antimicrobial property [17, 18]. Hence, the presence of these fatty acids in extracts of* Lenzites quercina* could contribute to its antimicrobial activity. Fatty acids possess potent antimicrobial property with broad spectrum activity against resistance organisms. The mechanism of antimicrobial effect by these acids is by affecting cell membrane and its components and interfering with mechanisms of virulence such as preventing biofilm formation and inhibiting the production of toxins and enzymes. Thus, the antimicrobial properties of free fatty acids can be exploited for the preservation of perishable products [19]. Hence,* Lenzites quercina* could be an alternative source of fatty acid with preservative effect in food and pharmaceutical industries.

The varying degree of inhibition zone observed for the extracts and purified fractions of* Lenzites quercina* may be due to the polarity of solvent. The solubility of secondary metabolites is highly dependent on the polarity of the solvents [12]. The inhibitory potentials of ethanolic extracts might be due to the higher solubility of active antimicrobial constituents. This conformed to Ayyasamy et al. [20] who also reported better inhibitory potential for ethanolic extract of* Pleurotus florida*. However, the inhibition zones obtained in this study deviated from Shikongo et al. [21] who observed no inhibition on* Staphylococcus aureus, Bacillus, Alcaligenes faecalis, *and* Proteus vulgaris* with ethanolic extracts of* Ganoderma lucidum* but benzene, chloroform, and ethyl acetate extracts gave better inhibition on the tested organisms.

The antimicrobial action of ethyl acetate extracts on tested microorganisms also deviated from the findings of Rajesh and Dhanasekaran [22] who reported more inhibition zones for ethyl acetate extracts of* Ganoderma* species.

The antimicrobial potential of extracts obtained from* Lenzites quercina* could be attributed to presence of the phytochemicals listed above. Flavonoids are known to enhance antimicrobial activity by creating complexes with extracellular soluble proteins and polypeptides in the cell wall of microorganisms, which will disrupt the function of cell membrane of microorganisms [23]. Antimicrobial property of tannins had been due to its ability to link amino acids in proteins, inactivating adhesions, enzymes, and transport proteins of cell membranes of microorganisms [24]. Saponins are also known to have cytotoxic effects with surfactant properties on cell membrane, which assist in destruction of invading microorganisms [25]. Hence, the presence of alkaloids in* Lenzites quercina *extracts greatly marked its inhibitory property, since it is a known phytochemical that possesses antimicrobial activity [26].

In spite of the selective therapeutic option acquired by pathogenic organisms due to endogenous gene responsible for antibiotic resistance, extracts of* Lenzites quercina* exhibited inhibition on some of these clinical isolates that are known to have developed resistance to antibiotics. The range of inhibition zones observed (4 mm to 26 mm) in this study conformed to Jonathan and Awotona [27] and Chowdhury et al. [28] who in their findings reported antimicrobial potential of some medicinal mushroom extracts with zones of inhibition between 3.7 mm and 20.3 mm.* Lenzites quercina* extracts exhibited inhibitory effect on typed culture; Al-Fatimi et al. [29] had also tested number of medicinal mushrooms from Yemen on* P. aeruginosa *(ATCC 27853) and* S. aureus *(ATCC 29213). Thus, many wild macrofungi had been reported to demonstrate potent antimicrobial activities [7]. Extracts of raw and fermented* Lenzites quercina* displayed inhibitory action on the tested isolates. This showed that the fermentation process does not affect the antimicrobial quality of* Lenzites quercina*.

## 5. Conclusion

It was generally observed that the fractions obtained from the extracts of* Lenzites quercina* were more effective against tested bacteria and fungi. This may be due to the almost pure nature of the bioactive agent in the fraction unlike the multiple compounds that are found in extracts of* Lenzites quercina*. Generally, extracts of* Lenzites quercina* displayed broad spectrum antimicrobial action and could therefore be considered as an option in the treatment of common diseases caused by pathogenic organisms.

## Figures and Tables

**Table 1:** Phytochemical constituents (mg/g) of extracts from *Lenzites quercina*.

Phytochemicals	RPP	REA	RET	FPP	FEA	FET
Alkaloids	0.0	15.3^b^ ± 0.03	21.2^c^ ± 0.11	0.0	14.4^a^ ± 0.01	20.7^c^ ± 0.50
Saponins	10.6^e^ ± 0.10	6.2^b^ ± 0.10	7.7^c^ ± 0.20	8.3^d^ ± 0.09	4.5^a^ ± 0.25	7.0^c^ ± 0.10
Flavonoids	1.92^b^ ± 0.0	0.45^a^ ± 0.0	7.10^d^ ± 0.0	0.41^a^ ± 0.0	0.38^a^ ± 0.0	5.4^c^ ± 0.0
Tannins	0.0	10.4^a^ ± 0.00	18.6^c^ ± 0.10	0.0	13.9^b^ ± 0.90	0.0
Phlobatannins	0.0	0.0	0.0	0.0	0.0	0.0
Steroids	12.8^d^ ± 0.20	9.9^b^ ± 0.05	12.0^c^ ± 0.05	0.0	0.0	6.1^a^ ± 0.10
Anthraquinones	0.0	0.0	1.9 ± 0.50	0.0	0.0	0.0
Terpenoids	1.45^a^ ± 0.40	0.0	24.8^e^ ± 0.20	5.0^c^ ± 0.06	2.8^b^ ± 0.01	17.2^d^ ± 0.60
Cardiac glycosides	6.3^a^ ± 0.02	5.7^a^ ± 0.06	12.06^d^ ± 0.08	9.3^b^ ± 0.10	0.0	10.0^c^ ± 0.3

Values are mean ± sd of replicates (*n* = 3). Values with the same alphabet along row are not significantly different at *P* = 0.05.

RPP: raw *Lenzites quercina* extracted with pet-ether, REA: raw *Lenzites quercina* extracted with ethyl acetate, RET: raw *Lenzites quercina* extracted with ethanol, FPP: fermented *Lenzites quercina* extracted with pet-ether, FEA: fermented *Lenzites quercina* extracted with ethyl acetate, and FET: fermented *Lenzites quercina* extracted with ethanol.

**Table 2:** Bioactive compounds in ethanolic extract of *Lenzites quercina*.

Number	Retention time	Peak area	Name of compounds	Molecular formula	Molecular weight
1	8.63	8.17	Caprylic acid	C_8_H_16_O_2_	144
2	11.20	6.65	n-Decanoic acid	C_10_H_20_O_2_	172
3	13.83	61.06	Dodecanoic acid	C_12_H_24_O_2_	200
4	14.14	9.22	Phosphoric acid tributyl ester	C_12_H_27_O_4_P	266
5	16.06	10.69	Tetradecanoic acid	C_14_H_28_O_2_	228
6	19.54	2.06	Stearic acid	C_18_H_36_O_2_	284
7	21.11	0.50	Methyl-11-octadecenoate	C_19_H_34_O_2_	296
8	22.21	1.13	Oleic acid	C_18_H_34_O_2_	282
9	26.18	0.51	4-Methyl-2-propyl-1-pentanol	C_9_H_20_O	144

**Table 3:** Zones of inhibition (mm) of raw and fermented extracts against indicator organisms at 50 mg/mL.

Tested isolates	RPP	REA	RET	FPP	FEA	FET	CPF	VAN/FLU
*S. aureus* (ATCC 29213)	0.0	0.0	9.0 ± 0.0	0.0	0.0	10.0 ± 0.0	15.0 ± 0.0	12.0 ± 0.0
*E. coli* (ATCC 35218)	12.0 ± 0.0	10.3 ± 0.5	12.7 ± 0.5	0.0	0.0	0.0	8.0 ± 0.0	10.0 ± 0.0
*P. aeruginosa* (ATCC 27853)	0.0	11.0 ± 0.0	13.0 ± 0.0	0.0	0.0	12.0 ± 0.0	0.0	17.0 ± 0.0
*S. flexneri* (ATCC 12022)	0.0	0.0	0.0	0.0	0.0	0.0	0.0	20.0 ± 0.0
*S. enterica* (ATCC 33458)	6.3 ± 0.0	10.0 ± 0.0	16.0 ± 0.0	10.7 ± 0.3	11.0 ± 0.0	14.7 ± 0.3	10 ± 0.0	18.3 ± 0.2
*B. subtilis* (ATCC 6633)	0.0	0.0	6.0 ± 0.0	0.0	14.0 ± 0.0	8.0 ± 0.0	0.0	14.0 ± 0.0
*E. aerogenes* (ATCC 13048)	16.2 ± 0.3	0.0	8.7 ± 0.6	10.3 ± 0.5	0.0	0.0	8.3 ± 0.6	12.0 ± 0.0
*S. flexneri*	12.0 ± 0.0	0.0	0.0	0.0	7.0 ± 0.0	12.0 ± 0.0	9.3 ± 0.5	12.0 ± 0.0
*S. typhi*	0.0	12.0 ± 0.0	8.0 ± 0.0	0.0	12.0 ± 0.0	0.0	0.0	9.0 ± 0.0
*P. aeruginosa*	0.0	0.0	9.0 ± 0.0	0.0	0.0	7.3 ± 0.2	0.0	10 ± 0.0
*B. cereus*	8.0 ± 0.0	0.0	11.0 ± 0.0	14.0 ± 0.0	0.0	9.0 ± 0.0	0.0	13.3 ± 0.3
*E. faecalis*	0.0	0.0	8.0 ± 0.0	0.0	10.0 ± 0.0	13.0 ± 0.0	9.3 ± 0.2	11.3 ± 0.5
*E. coli*	10.7 ± 0.5	0.0	0.0	14.3 ± 0.6	0.0	12.0 ± 0.0	13.0 ± 0.0	0.0
*S. aureus*	8.7 ± 0.5	0.0	10.7 ± 0.3	5.0 ± 0.0	11.7 ± 0.6	0.0	0.0	8.3 ± 0.0
MRSA-1	0.0	0.0	14.0 ± 0.0	0.0	0.0	11.3 ± 0.2	10.0 ± 0.4	10 ± 0.0
MRSA-2	0.0	0.0	0.0	0.0	0.0	0.0	0.0	6.0 ± 0.0
MRSA-3	0.0	0.0	0.0	0.0	0.0	0.0	0.0	11.3 ± 0.2
MRSA-4	0.0	4.0 ± 0.0	8.0 ± 0.2	0.0	0.0	0.0	0.0	6.5 ± 0.02
MRSA-5	0.0	6.0 ± 0.0	18.0 ± 0.0	0.0	6.0 ± 0.0	13.0 ± 0.0	7.0 ± 0.0	8.0 ± 0.01
MRSA-6	0.0	0.0	10.1 ± 0.0	0.0	0.0	0.0	0.0	6.0 ± 0.0
^*∗*^ *C*. *albicans*	0.0	0.0	11.3 ± 0.0	0.0	0.0	18.0 ± 0.0	NT	20.0 ± 0.0
^*∗*^ *A. niger*	8.0 ± 0.0	0.0	17.2 ± 0.1	0.0	0.0	0.0	NT	18.0 ± 0.0
^*∗*^ *A. flavus*	0.0	0.0	0.0	0.0	0.0	13.0 ± 0.0	NT	8.2 ± 0.11

Values are mean ± sd of replicates (*n* = 3).

RPP: raw *Lenzites quercina* extracted with pet-ether, REA: raw *Lenzites quercina* extracted with ethyl acetate, RET: raw *Lenzites quercina* extracted with ethanol, FPP: fermented *Lenzites quercina* extracted with pet-ether, FEA: fermented *Lenzites quercina* extracted with ethyl acetate, FET: fermented *Lenzites quercina* extracted with ethanol, CPF: ciprofloxacin (10 *μ*g), VAN: vancomycin (30 *μ*g), and NT: not tested; ^*∗*^fungi are tested against FLU, fluconazole (20 mg), and 0.0, no zone of inhibition.

**Table 4:** Zones of inhibition of purified fractions against tested organisms at 2.0 mg/mL.

Indicator organisms	RPE	RCL	RMT	FPE
*S*. *aureus* (ATCC 29213)	18.0 ± 0.0	0.0	14.0 ± 0.0	0.0
*E. coli* (ATCC 35218)	15.0 ± 0.0	0.0	11.1 ± 0.2	0.0
*P. aeruginosa* (ATCC 27853)	0.0	11.0 ± 0.0	17.0 ± 0.0	0.0
*S. flexneri* (ATCC 12022)	0.0	0.0	8.0 ± 0.0	0.0
*S. enterica* (ATCC 33458)	6.3 ± 0.0	18.0 ± 0.0	0.0	0.0
*B. subtilis* (ATCC 6633)	0.0	0.0	10.0 ± 0.0	6.0 ± 0.0
*E. aerogenes* (ATCC 13043)	21.5 ± 0.02	0.0	0.0	8.0 ± 0.2
*Shigella flexneri*	0.0	0.0	10.0 ± 0.0	0.0
*S. typhi*	26.0 ± 0.0	0.0	18.0 ± 0.0	0.0
*Pseudomonas aeruginosa*	0.0	0.0	16.2 ± 0.6	0.0
*Bacillus cereus*	0.0	0.0	23.0 ± 0.0	11.0 ± 0.3
*Enterococci faecalis*	0.0	0.0	20.0 ± 0.0	0.0
*E. coli*	13.3 ± 0.4	0.0	0.0	0.0
MRSA-1	0.0	0.0	19.0 ± 0.0	0.0
MRSA-2	0.0	0.0	0.0	0.0
MRSA-3	0.0	0.0	0.0	0.0
MRSA-4	10.2 ± 0.2	0.0	0.0	9.1 ± 0.23
MRSA-5	0.0	0.0	15.1 ± 0.3	0.0
MRSA-6	0.0	0.0	13.2 ± 0.1	0.0
*S. aureus*	22.0 ± 0.0	0.0	16.0 ± 0.0	0.0
*Candida albicans*	13.1 ± 0.3	0.0	0.0	0.0
*Aspergillus niger*	20.0 ± 0.0	0.0	0.0	0.0
*Aspergillus flavus*	17.0 ± 0.0	0.0	0.0	0.0

Values are mean ± sd of replicates (*n* = 3).

RPE: pet-ether fraction, RCL: chloroform fractions, RMT: methanol fraction, FPE: pet-ether fraction (fermented *L. quercina*), and 0.0: no zone of inhibition at 2.0 mg/mL.

**Table 5:** Minimum inhibitory concentration (MIC) of *Lenzites quercina* extracts (mg/mL) against tested organisms.

Microorganisms	RPP	REA	RET	FPP	FEA	FET
*S. aureus* (ATCC 29213)	0.0	0.0	3.125	0.0	0.0	3.125
*E. coli* (ATCC 35218)	25.0	12.5	6.25	0.0	0.0	0.0
*P. aeruginosa* (ATCC 27853)	0.0	25.0	12.5	0.0	0.0	12.5
*S. flexneri* (ATCC 12022)	0.0	0.0	0.0	0.0	0.0	0.0
*S. enterica* (ATCC 33458)	12.5	12.5	25.0	25.0	25.0	6.25
*B. subtilis* (ATCC 6633)	0.0	0.0	3.125	0.0	6.25	6.25
*E. aerogenes* (ATCC 13043)	6.25	0.0	6.25	25.0	0.0	0.0
*S. flexneri*	12.5	0.0	0.0	0.0	0.0	25.0
*S. typhi*	0.0	12.5	6.25	0.0	12.5	0.0
*P. aeruginosa*	0.0	0.0	12.5	0.0	0.0	50.0
*B. cereus*	6.25	0.0	12.5	3.125	0.0	6.25
*E. faecalis*	0.0	0.0	50.0	0.0	0.0	12.5
*E. coli*	50.0	0.0	0.0	6.25	0.0	25.0
*S. aureus*	12.5	0.0	6.25	3.125	25.0	0.0
MRSA-1	0.0	0.0	25.0	0.0	0.0	25.0
MRSA-2	0.0	0.0	0.0	0.0	0.0	0.0
MRSA-3	0.0	0.0	0.0	0.0	0.0	0.0
MRSA-4	0.0	12.5	6.25	0.0	0.0	0.0
MRSA-5	0.0	25.0	25.0	0.0	25.0	6.25
MRSA-6	0.0	0.0	12.5	0.0	0.0	0.0
*Candida albicans*	0.0	0.0	6.25	0.0	0.0	0.0
*Aspergillus niger*	25.0	0.0	12.5	0.0	0.0	0.0
*Aspergillus flavus*	0.0	0.0	0.0	0.0	0.0	0.0

Values are mean of replicates (*n* = 3).

RPP: raw *Lenzites quercina* extracted with pet-ether, REA: raw *Lenzites quercina* extracted with ethyl acetate, RET: raw *Lenzites quercina* extracted with ethanol, FPP: fermented *Lenzites quercina* extracted with pet-ether, FEA: fermented *Lenzites quercina* extracted with ethyl acetate, FET: fermented *Lenzites quercina* extracted with ethanol, and 0.0: no zone of inhibition at 50 mg/mL.

**Table 6:** Minimum bactericidal concentration (MBC) of *Lenzites quercina* extracts (mg/mL) against tested organisms.

Microorganisms	RPP	REA	RET	FPP	FEA	FET
*S. aureus* (ATCC 29213)	0.0	0.0	12.5	0.0	0.0	25.0
*E. coli* (ATCC 35218)	25.0	12.5	6.25	0.0	0.0	0.0
*P. aeruginosa* (ATCC 27853)	0.0	50	12.5	0.0	0.0	12.5
*S. flexneri* (ATCC 12022)	0.0	0.0	0.0	0.0	0.0	0.0
*S. enterica* (ATCC 33458)	12.5	25	50	50.0	25.0	6.25
*B. subtilis* (ATCC 6633)	0.0	0.0	6.25	0.0	12.5	12.5
*E. aerogenes* (ATCC 13043)	12.5	0.0	6.25	25.0	0.0	0.0
*S. flexneri*	25	0.0	0.0	0.0	0.0	25.0
*S. typhi*	0.0	12.5	12.5	0.0	12.5	0.0
*P. aeruginosa*	0.0	0.0	50.0	0.0	0.0	50.0
*B. cereus*	50.0	0.0	12.5	6.25	0.0	6.25
*E. faecalis*	0.0	0.0	50.0	0.0	0.0	12.5
*E. coli*	50	0.0	0.0	6.25	0.0	25.0
*S. aureus*	12.5	0.0	12.5	12.5	50.0	0.0
MRSA-1	0.0	0.0	100.0	0.0	0.0	50.0
MRSA-2	0.0	0.0	0.0	0.0	0.0	0.0
MRSA-3	0.0	0.0	0.0	0.0	0.0	0.0
MRSA-4	0.0	25.0	25.0	0.0	0.0	0.0
MRSA-5	0.0	25.0	12.5	0.0	50.0	12.5
MRSA-6	0.0	0.0	25.0	0.0	0.0	0.0
*Candida albicans*	0.0	0.0	50.0	0.0	0.0	0.0
*Aspergillus niger*	25.0	0.0	25.0	0.0	0.0	0.0
*Aspergillus flavus*	0.0	0.0	0.0	0.0	0.0	0.0

Values are mean of replicates (*n* = 3).

RPP: raw *Lenzites quercina* extracted with pet-ether, REA: raw *Lenzites quercina* extracted with ethyl acetate, RET: raw *Lenzites quercina* extracted with ethanol, FPP: fermented *Lenzites quercina* extracted with pet-ether, FEA: fermented *Lenzites quercina* extracted with ethyl acetate, FET: fermented *Lenzites quercina* extracted with ethanol, and 0.0: no zone of inhibition at 50 mg/mL.
